# Patients’ Attitude toward Less Frequent Surveillance of Low-Risk Pancreatic Cysts: A Multicenter European Cohort Study

**DOI:** 10.1177/0272989X251352750

**Published:** 2025-08-03

**Authors:** Marloes L. J. A. Sprij, Inge M. C. M. de Kok, Daan D. Nieboer, Gabriele Capurso, Jihane Meziani, Mattheus C. B. Wielenga, Mirjam C. M. van der Ende, Marianne E. Smits, Riccardo Casadei, Matthijs P. Schwartz, Frederike G. I. van Vilsteren, Chantal Hoge, Rutger Quispel, Pieter Honkoop, Laurens A. van der Waaij, Gemma Rossi, Adriaan C. I. T. L. Tan, Marco J. Bruno, Djuna L. Cahen

**Affiliations:** Department of Gastroenterology and Hepatology, Erasmus University Medical Center, Rotterdam, The Netherlands; Department of Public Health, Erasmus University Medical Center, Rotterdam, The Netherlands; Department of Public Health, Erasmus University Medical Center, Rotterdam, The Netherlands; Department of Public Health, Erasmus University Medical Center, Rotterdam, The Netherlands; Department of Gastroenterology and Hepatology, Vita Salute San Raffaele University, Milan, Italy; Department of Gastroenterology and Hepatology, Erasmus University Medical Center, Rotterdam, The Netherlands; Department of Gastroenterology & Hepatology, Amsterdam UMC, Amsterdam, The Netherlands; Department of Gastroenterology and Hepatology, Catharina Hospital, Eindhoven, The Netherlands; Department of Gastroenterology & Hepatology, Tergooi, Hilversum, The Netherlands; Department of Surgery, Bologna University, Bologna, Italy; Department of Gastroenterology & Hepatology, Meander Medical Center, Amersfoort, The Netherlands; Department of Gastroenterology & Hepatology, University Medical Center Groningen, Groningen, The Netherlands; Department of Gastroenterology & Hepatology, Maastricht University Medical Center, Maastricht, The Netherlands; Department of Gastroenterology & Hepatology, Reinier de Graaf hospital, Delft, The Netherlands; Department of Gastroenterology & Hepatology, Albert Schweitzer Hospital, Dordrecht, The Netherlands; Department of Gastroenterology & Hepatology, Martini Hospital, Groningen, The Netherlands; Pancreato-Biliary Endoscopy and Endosonography Division, Pancreas Translational and Clinical Research Center, San Raffaele Scientific Institute IRCCS, Vita Salute San Raffaele University, Milan, Italy; Department of Gastroenterology & Hepatology, Canisius Wilhelmina hospital, Nijmegen, The Netherlands; Department of Gastroenterology and Hepatology, Erasmus University Medical Center, Rotterdam, The Netherlands; Department of Gastroenterology and Hepatology, Erasmus University Medical Center, Rotterdam, The Netherlands

**Keywords:** Pancreatic cyst surveillance, low-risk pancreatic cysts, patient atttitudes, surveillance frequency

## Abstract

**Background:**

Recent studies show that low-risk pancreatic cysts may require less frequent monitoring. Future guidelines will likely adapt their recommendations accordingly. Our goal was to explore the willingness of individuals with a low-risk pancreatic cyst to undergo less frequent surveillance and to identify associated characteristics with such willingness.

**Methods:**

This is a side study of the international PACYFIC study, which prospectively collects data on cyst surveillance, including questionnaires to assess participants’ attitude toward surveillance. Individuals with low-risk cysts at baseline, without given standardized information by the study protocol, were enrolled. Their responses to the baseline question, “Would you prefer less frequent surveillance? Yes/No,” were correlated with baseline characteristics using multivariable logistic regression, namely, age, country of residence, symptoms, medical and family history, time since first cyst detection, and Hospital Anxiety Depression Scale score.

**Results:**

Of the 215 participants included from the Netherlands (*n* = 185) and Italy (*n* = 30), only 47 (22%) were willing to undergo less surveillance. Characteristics positively associated with this willingness were older age (odds ratio [OR] 1.87 per 10 y, 95% confidence interval [CI]: 1.15–3.04) and Italian residency (OR 16.35, 95% CI: 5.65–47.31). A medical history of cancer was negatively associated (OR 0.28, 95% CI: 0.09–0.90). No other associations were observed.

**Conclusion:**

Most participants appear unwilling to undergo less frequent cyst surveillance. Older age and residing in Italy were associated with a greater willingness toward less rigorous surveillance, while a history of cancer did the opposite. Identifying which individuals are hesitant to undergo less frequent surveillance may help clinicians tailor their counseling and can support implementation of future guideline with fewer surveillance recommendations.

**Highlights:**

- Most low-risk individuals were reluctant toward less frequent pancreatic cyst surveillance.
- Older age and residency in Italy were associated with a higher willingness.
- A medical history of cancer was associated with an unwillingness.
- Standardized patient information may increase the willingness, but such information has yet to be developed.

## Introduction

Pancreatic cysts are increasingly detected due to the growing use of high-quality cross-sectional imaging.^1,2^ It is estimated that approximately half of these cysts are mucinous and bear malignant potential.^3,4^ Depending on the presence of certain risk factors, referred to as worrisome features (WFs) and high-risk stigmata (HRS), international guidelines advise surgical removal or lifelong annual surveillance by radiologic imaging.^5–7^

Recent studies suggest that cysts without WFs or HRS, the so-called low-risk cysts, carry a considerably lower cancer risk than originally believed.^8–10^ According to a meta-analysis by Chhoda et al.,^11^ the risk of advanced neoplasia in a low-risk cyst is a mere 1%, in contrast to the 85 to 25% risk reported in earlier surgical series.^12,13^ This implies that less frequent or even cessation of surveillance might suffice for these cysts, and future guidelines will likely adapt their recommendations accordingly.^14^

Implementation of less rigorous guidelines may prove challenging due to patients’ general reluctance toward less-intensive surveillance.^15–17^ Understanding an individuals’ attitude toward surveillance will enable clinicians to tailor their communication strategies and information delivery and thus can improve informed-decision making. As a first step, this study aims to explore the current willingness of individuals with low-risk pancreatic cysts to undergo less frequent surveillance and to identify patient characteristics associated with such willingness. In line with the principles of “pragmatic trials,”^18^ we sought to capture patients’ unmodified attitudes toward reduced surveillance based solely on the information provided by their treating physician.

## Methods

### Study Design

The current study is a side study of the Pancreatic Cyst Follow-up, an International Collaboration (PACYFIC) registry. Since 2015, this multicenter observational cohort study has evaluated the yield of pancreatic cyst surveillance. It includes individuals with neoplastic and undefined pancreatic cysts, either newly or previously diagnosed or operated upon, who are being followed at the discretion of their treating physician. Participants are informed of their diagnosis and risk status by their treating physician. The PACYFIC study does not provide additional patient information. Similarly, in this side study, patients did not receive supplemental information from the study team regarding their risk status or the rationale behind risk-based surveillance. This approach is consistent with the design of “pragmatic trials,”^18^ which aim to replicate real-world clinical settings. For example, providing detailed risk information in studies of risk perception may lead to an overestimation of patients’ actual understanding. Information regarding the characteristics of the participants and cysts is prospectively recorded in an online case record form (www.pacyfic.net) by the treating physicians. In addition, participants complete questionnaires by email regarding their history and attitude toward surveillance. At the time of this side study, 2 countries from North America and 13 countries from Europe have participated (Appendix A, Supplementary Table S1).

The study was approved by the institutional review board of the Erasmus Medical Center and was executed according to the principles of the Declaration of Helsinki and the Declaration of Taipei (2016) and in accordance with the Medical Research Involving Human Subjects Act (WMO). Participants were enrolled after they provided their written informed consent.

### PACYFIC Questionnaire

The PACYFIC questionnaire (Appendix B) consists of 3 parts: 1) questions related to lifestyle and personal and family history, 2) inquiries about the participants’ attitude toward surveillance, and 3) the Hospital Anxiety and Depression Scale (HADS) score. The HADS is a validated tool that assesses possible symptoms of anxiety (HADS-A) and depression (HADS-D). It consists of 14 questions divided into 2 subscales, 1 for anxiety and 1 for depression, each scored from 0 to 21. A score of >8 reflects a possible anxiety disorder or depression.^19^

### Participant and Country Selection

PACYFIC participants with low-risk cysts at baseline (without WFs and/or HRS according to the European guidelines^5^) who had completed the baseline questionnaire were included in the present study. Countries with fewer than 10 participants were excluded.

### Variables and Outcome

Collected participant characteristics included gender, age, race, country of residency, history of cancer (i.e., breast, colon, lung, melanoma, prostate, other), family history of pancreatic cancer, gastroenterological symptoms (i.e., abdominal pain, weight loss, fatigue) and the HADS score. Cyst characteristics included the size of the largest cyst, diagnosis (i.e., branch-duct intraductal papillary mucinous neoplasm [BD-IPMN], mucinous cystic neoplasm, unspecified cyst) according to the treating physician, and years since first detection of the cyst. All variables, except the HADS score, were collected by the physician through the electronic case record. The HADS score was obtained through the patient self-reported questionnaire. The primary outcome was the response to the question, “Would you prefer your cyst to be checked less frequently?” The 4-point scale answers were dichotomized to allow for multivariable dichotomous logistic regression analysis. The answers *somewhat*, *rather*, and *very much* were considered as willing, and the answer *not at all* was considered as unwilling.

### Imputation and Statistical Analysis

The means with standard deviation or medians with range were calculated depending on the distribution of the data. Categorical variables were expressed as frequencies and percentages. Outliers were identified and verified by contacting the principal investigator of the hospital, who cross-referenced the data with the original patient chart. Subsequently, an analysis with and without outliers was conducted to assess their potential impact. If a significant change in effect was observed, the outliers were excluded from the analysis.

Substantial missing values (>10%) were investigated for clustering within a specific subgroup or variable. If no clustering was identified, the missing values were imputed 5 times using chained equations. Statistical analyses were performed on each of the imputed datasets, and subsequently results were pooled using Rubin rules.

Due to the limited number of inclusions, we carefully selected the variables to examine in the regression analysis. In the absence of existing literature to guide variable selection, emphasis was placed on variables we deemed relevant to participants’ knowledge and to their attitude toward surveillance reduction. In addition, we focused on participant characteristics, such as symptoms or age, that could potentially influence their willingness.

Univariable and multivariable dichotomous logistic regression analysis with backward selection was conducted to investigate independent variables that were associated with willingness to undergo less surveillance. During the backward selection process, the least significant variables were stepwise eliminated until all variables with a *P* value ≥0.1 were excluded from the multivariable model. Possible interaction terms were tested and included in the model when they had a significant contribution. A *P* value <0.05 was considered significant. In addition, ordinal logistic regression analyses were performed and presented in the supplementary materials. Data analyses were performed using IBM SPSS Statistics (version 28.0.1.0).

## Results

### Study Population

**Figure 1:**
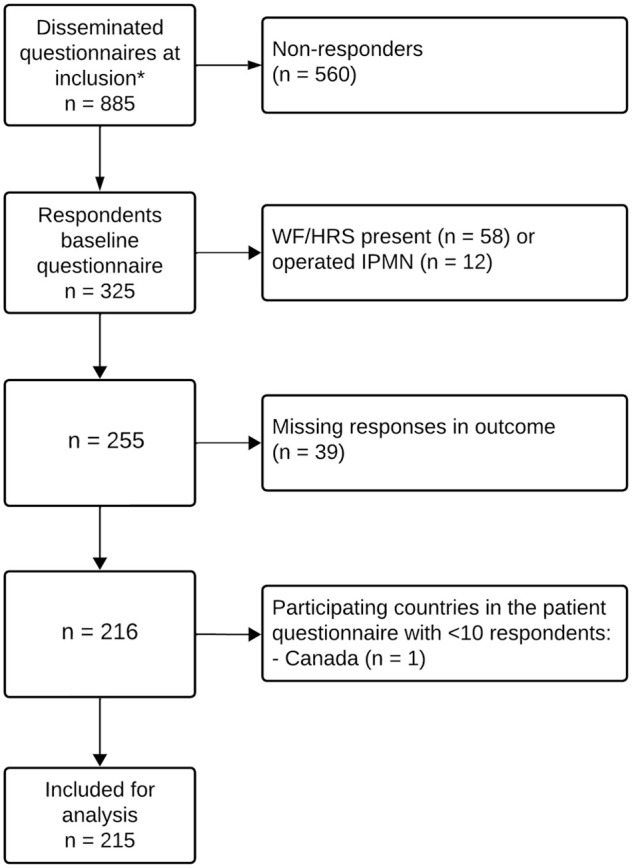
Flowchart of participant selection. HRS, high-risk stigmata; WF, worrisome feature. *Participating countries that disseminated the PACYFIC questionnaire: Italy, the Netherlands, Germany, Hungary, the United States, and Canada.

By June 2023, 6 countries were actively participating in the questionnaire. A total of 885 participants had provided their email addresses, of whom 327 had returned the baseline questionnaire, leading to a response rate of 37%. After excluding those with missing outcomes and applying the exclusion criteria, 215 respondents residing in the Netherlands (*n* = 185, 86%) and Italy (*n* = 30, 14%) were eligible for analysis (Figure 1). Table 1 shows their baseline characteristics. The median age was 68 y, with 137 (64%) identifying as female and most being Caucasian (*n* = 202; 94%). The median number of years since first cyst detection was 2 (range 0–15). Participants were predominantly diagnosed with BD-IPMNs (*n* = 186, 87%), followed by unspecified cysts (*n* = 23, 11%). The median size of the participants’ largest cyst in our study population was 15 (range 3–39) mm. According to the HADS scores, a possible anxiety disorder was present in 39 (18%) and possible depression in 20 (9%).

**Table 1:** Baseline Characteristics of the Included Respondents and the Nonparticipating Individuals, Who Either Did Not Participate due to their Country’s Nonparticipation in the Questionnaire or as Nonrespondents

	Respondents, *n* = 215 (%)	Missing (%)	Nonparticipant, *n* = 1,574 (%)	Missing (%)
Participant characteristics				
Female	137 (64)	0	1,007 (64)	0
Age at inclusion, y, median (range)	68 (32–83)	0	69 (24–97)	0
Race		4 (2)		94 (6)
Caucasian	202 (94)		1,427 (91)	
Other	9 (4)		53 (3)	
Country of residency		0		0
Netherlands	185 (86)		691 (44)	
Italy	30 (14)		273 (17)	
Other	0		610 (39)	
Family history of pancreatic cancer	20 (9)	46 (21)	115 (7)	203 (13)
Medical history of cancer^a^	49 (23)	0	278 (18)	1 (0)
Gastroenterological symptoms present^b^	28 (13)	0	236 (15)	0
HADS-Anxiety score ≥8^c^	39 (18)	16 (7)		
HADS-Depression score ≥8^c^	20 (9)	11 (5)		
Cyst characteristics				
Diagnosis cyst		0		0
BD-IPMN	186 (87)		1,379 (88)	
Unspecified cyst	23 (11)		157 (10)	
MCN	3 (1)		18 (1)	
Other	3 (1)		20 (1)	
Largest cyst size in 1 individual, mm, median (range)	15 (3–39)	0	15 (2–39)	19 (1)
Years since first cyst detection, median (range)	2 (0–15)	0	1 (0–18)	0

BD-IPMN, branch-duct intraductal papillary mucinous neoplasm; HADS, Hospital Anxiety and Depression Scale; MCN, mucinous cystic neoplasm.

a History with cancer included breast, colon, lung, melanoma, prostate, and other.

b Symptoms included abdominal pain, weight loss, fatigue, and other.

c A score ≥8 reflects a possible anxiety disorder or depression.

### Willingness to Undergo Less Frequent Surveillance

In response to the 4-point scale question, “Would you prefer your cyst to be checked less frequently?” 168 participants (78%) chose the negative answer (*not at all*), whereas 47 participants (22%) expressed a more positive attitude toward less frequent surveillance (*somewhat*, *n* = 23; *rather*, *n* = 23; *very much*, *n* = 1). Of the participants, 27.3% of those ≥70 y of age were willing to undergo less surveillance, compared with 18.1% of individuals younger than 70 y. In the univariable analysis, older age, residing in Italy, and having higher HADS-A and -D scores were significantly associated with a willingness to undergo less frequent surveillance (Table 2). In the dichotomous multivariable analysis, only older age (odds ratio [OR] 1.87, 95% confidence interval [CI]: 1.15–3.04) and residing in Italy (OR 16.35, 95% CI: 5.65–47.31) remained positively associated. A history of cancer did not appear associated in the univariable model, but in the multivariable analysis, it was associated with reluctance to undergo less frequent surveillance (OR 0.28, 95% CI: 0.09–0.90). A family history of pancreatic cancer and years since first cyst diagnosis did not affect the attitude toward willingness to undergo less frequent surveillance. Both the univariable and multivariable ordinal logistic regression analyses showed similar effect sizes (Appendix C, Supplementary Table S2).

**Table 2:** Univariable and Multivariable Logistic Regression Analysis for Possible Associated Characteristics with a Willingness to Undergo Less Frequent Surveillance^a^

	Univariable		Multivariable	
	Odds Ratio	95% CI	Odds Ratio	95% CI
Age (per 10-yincrease)	**1.93**	**1.26–2.96**	**1.87**	**1.15–3.04**
Gender				
Female	Ref		NA	
Male	1.41	0.73–2.72	NA	
Country of residency				
Netherlands	Ref		Ref	
Italy	**17.60**	**7.07–43.83**	**16.35**	**5.65–47.31**
Family history of pancreatic cancer				
Not present	Ref		NA	
Present	0.44	0.08 - 2.32	NA	
Medical history of cancer^b^				
Not present	Ref		Ref	
Present	0.64	0.28–1.47	**0.28**	**0.09–0.90**
Gastroenterological symptoms^c^				
Not present	Ref		Ref	
Present	0.39	0.11–1.35	0.16	0.03–1.04
Years since first cyst detection (per year increase)	1.06	0.95–1.19	NA	
HADS-Anxiety	**1.13**	**1.04–1.23**	NA	
HADS-Depression	**1.16**	**1.05–1.28**	1.12	0.98–1.27

CI, confidence interval; HADS, Hospital Anxiety and Depression Scale; NA, not applied in the model after applying the backward selection method; Ref, reference. Values in bold indicate statistical significance (*P* value <0.05)

a A backward selection method was used in the multivariable logistic regression.

b History with cancer included breast, colon, lung, melanoma, prostate, and other.

c Symptoms included abdominal pain, weight loss, fatigue, and other.

## Discussion

In this study, we explored the current willingness to undergo less surveillance in a low-risk pancreatic cyst surveillance population. Without providing additional information on the patients’ risk status and the rationale behind risk-based surveillance, the vast majority indicated an unwillingness to reduce the frequency of surveillance. Residency in Italy and older age were associated with a more favorable attitude toward reduced surveillance, while individuals with a history of cancer were more reluctant toward less surveillance. For the first time, the most recent international guideline recommends a reduction in surveillance frequency for low-risk individuals.^14^ This highlights the importance of gaining more knowledge about individuals’ attitudes toward these changes.

This study marks the first step toward gaining insight into participants’ current preferences regarding surveillance frequency in a low-risk cyst population. Our finding that only 22% of participants were willing to undergo less frequent surveillance aligns with the results of Koitsalu et al.,^17^ who reported 27%. This is not surprising as individuals generally seek reassurance. However, this indicates that implementation of future guidelines with reduced surveillance recommendations might be difficult. A solution to enhance implementation and increase willingness could be to utilize standardized information explaining the rationale behind the surveillance strategy. Studies that used standardized information regarding risk-based screening reported a much higher willingness of up to 59%. Participants were also more reassured and comfortable with the screening frequency.^16,20,21^ Moreover, other qualitative studies show that people are open to risk-based screening, particularly when well-informed.^22,23^ This emphasizes the importance of standardized and tailored information, but for cyst surveillance, such information has yet to be developed.

It was notable that many characteristics were not significantly associated, suggesting that it may be hard to identify a group that is willing to undergo less frequent surveillance. Surprisingly, the time since first cyst detection did not influence the attitude, although we hypothesized that those who had undergone surveillance for many years would be more unwilling. Also, family history of pancreatic cancer was similarly not associated. We presumed that participants would perceive themselves at higher risk for pancreatic cancer or, due to their experience with this deadly disease, prefer the reassurance of their usual surveillance frequency over a reduced one.^24–26^ It is possible that the degree and/or the number of affected relatives may influence this hypothesis; however, data were unavailable to investigate this further. Moreover, we expected that the HADS scores would have an impact on the willingness of reduced surveillance frequency, as studies reported that individuals with generally higher depression and anxiety scores are less likely to undergo screening.^27,28^ Our univariable analysis shows this trend. However, the effect dissipates in the multivariable analysis, suggesting that the true effect may either lie elsewhere or be attributed to a reduced statistical power due to the inclusion of numerous variables in the model.

Residing in Italy, as opposed to in the Netherlands, showed the strongest association with a willingness to undergo less frequent surveillance. The ease of implementing future guidelines may therefore be country dependent. We hypothesized several possible explanations. It might be that the Dutch desire more influence and control over their surveillance intensity compared with the Italians, given their known preference for having a choice in health care.^29^ Also, the doctor–patient communication in Italy tends to be more affective compared with the Dutch.^30^ This potentially fosters better doctor–patient relationships, resulting in improved patient adherence.^31–34^ Perhaps this also leads to an increased acceptance if the patient’s treating physician mentions a reduction in surveillance frequency. Lastly, access to the Italian health care system may involve other or more barriers compared with in the Netherlands. Two Italian studies highlighted the excessive waiting times for public health care and imaging modalities as major barriers.^35,36^ Consequently, an increasing number of Italian residents turn to private health care, where they must bear the full cost themselves.^36^ This might lead to Italian participants opting for less frequent surveillance. Nonetheless, more in-depth studies are needed to explore the reasons behind the association of Italian residence and the willingness to undergo less surveillance.

Not surprisingly, older age appeared to be associated with a willingness to undergo less frequent surveillance. This result aligns with other cancer screening studies that demonstrated age-related associations with less frequent screening preferences, lower attendance rates, or even cessation of screening.^37–39^ Moreover, Jansen et al.^40^ found that age is the most influential factor for older adults on their screening choices, independent of their life expectancy, quality of life, or physician recommendation. Previously reported reasons among the elderly include “having lived a good life,” not wanting to risk their current quality of life for treatment of the disease, or the risk associated with the tests.^39^

Lastly, individuals with a history of cancer were more reluctant to the idea of reduced surveillance. Understandably, such an experience often leads to more psychological distress or uncertainty,^41–43^ resulting in a stronger urge for reassurance. Another explanation might be that surveillance visits are less inconvenient, or normalized, for individuals with a history of cancer, given their regular hospital visits for cancer-related controls. Nevertheless, further studies are needed to explore these hypotheses.

According to the principles of pragmatic trials,^18^ the strength of this study lies in the exploration of patients’ willingness under real-life conditions, providing valuable insights into their preferences should a less frequent surveillance program be proposed. This study also has several limitations. The results should be interpreted with caution due to the relatively small study population, particularly regarding the identified significant variables. Larger sample sizes are needed to validate our results. Second, the generalizability of our findings may be limited, as they pertain specifically to a population already under surveillance. Newly diagnosed individuals may be more willing to undergo less surveillance compared with those who have been monitored for several years and have grown accustomed to it. Also, the extent to which participants were informed by their treating physicians or other sources (e.g., internet, social circle) is unclear. This may influence the willingness to undergo less surveillance. However, the general reluctance among participants suggests a need for better information provision. Furthermore, this study did not evaluate the participants’ motives behind preferring reduced surveillance as this was a preliminary study. This highlights the need for additional qualitative studies. Also, comorbidities and performance status, potential important variables influencing the willingness, were unknown. Lastly, the response rate was low to moderate (37%), potentially leading to a selection bias. However, we consider this unlikely, as the demographics of the nonparticipants were comparable to the participants.

## Conclusion

Currently, most individuals with a low-risk pancreatic cyst are unwilling to undergo less frequent surveillance. This might suggest potential challenges in implementing less rigorous surveillance guidelines in the future. Older age and residing in Italy were associated with greater acceptance of reduced surveillance frequency, while a history of cancer had the opposite effect. Identifying individuals’ attitudes toward reducing surveillance may help clinicians tailor their counseling and information delivery, resulting in better informed decision-making and smoother implementation of new guidelines. Offering standardized information that explains the rationale behind the reduced surveillance strategy will likely help in this process, but such information has yet to be developed. Further research is needed to understand the factors influencing participants’ surveillance preferences and the type of information they wish to receive.

## Supplemental Material

sj-docx-1-mdm-10.1177_0272989X251352750 – Supplemental material for Patients’ Attitude toward Less Frequent Surveillance of Low-Risk Pancreatic Cysts: A Multicenter European Cohort Study Supplemental material, sj-docx-1-mdm-10.1177_0272989X251352750 for Patients’ Attitude toward Less Frequent Surveillance of Low-Risk Pancreatic Cysts: A Multicenter European Cohort Study by Marloes L. J. A. Sprij, Inge M. C. M. de Kok, Daan D. Nieboer, Gabriele Capurso, Jihane Meziani, Mattheus C. B. Wielenga, Mirjam C. M. van der Ende, Marianne E. Smits, Riccardo Casadei, Matthijs P. Schwartz, Frederike G. I. van Vilsteren, Chantal Hoge, Rutger Quispel, Pieter Honkoop, Laurens A. van der Waaij, Gemma Rossi, Adriaan C. I. T. L. Tan, Marco J. Bruno and Djuna L. Cahen in Medical Decision Making
