# Supplementary material for: Patients’ Attitude toward Less Frequent Surveillance of Low-Risk Pancreatic Cysts: A Multicenter European Cohort Study
Source: Med Decis Making. 2025 Aug 3;45(7):904–12. doi: 10.1177/0272989X251352750 (PMC12413503; doi:10.1177/0272989X251352750)
Supplement: sj-docx-1-mdm-10.1177_0272989X251352750 – Supplemental material for Patients’ Attitude toward Less Frequent Surveillance of Low-Risk Pancreatic Cysts: A Multicenter European Cohort Study [file sj-docx-1-mdm-10.1177_0272989X251352750.docx]

# Appendix A

**Table S1**. Participating countries in the PACYFIC-study as of June 2023. Listed in alphabetic order.

| **Country** | |
| --- | --- |
| 1. | Belgium |
| 2. | Canada |
| 3. | Finland |
| 4. | Germany |
| 5. | Hungary |
| 6. | Italy |
| 7. | Ireland |
| 8. | Latvia |
| 9. | Malta |
| 10. | Poland |
| 11. | Romania |
| 12. | Serbia |
| 13. | Spain |
| 14. | The Netherlands |
| 15. | United States |

# Appendix B

**PACYFIC study**

- Baseline patient questionnaire -

**Instructions and additional information**

Thank you for participating in the PACYFIC study patient questionnaire.

This questionnaires will evaluate how you, as a patient, experience pancreatic cyst surveillance. The questionnaire also includes several questions regarding your background, lifestyle, and medical history. Subsequently, you will receive a standard questionnaire after each follow-up visit.

We expect it will take you 5 to 15 minutes to fill out the answers. Please read the instructions carefully before answering a question. We kindly ask you to fill out all questions, even if they seem unimportant or irrelevant to you. If you can skip a question, this will be explicitly mentioned.

There are no right or wrong answers. We are only interested in your personal opinion. Your answers will be handled and stored anonymously. We will never contact you regarding the answers you have given. If you have any questions or concerns, please don’t hesitate to contact the study coordinator (see contact information on the front page of this questionnaire) or your treating physician.

We thank you for your cooperation,

The PACYFIC research team.

I. **These first questions concern your personal background, lifestyle, and medical history.**

***Please fill out your details below.***

1. Are your being treated for Diabetes?

□ No ***Continue to question 2***

□ Yes ***Continue to question 1a***

1a. Since what year?___________________­

1b. Do you use Insulin?

□ No ***Continue to question 2***

□ Yes ***Continue to question 1c***

1c. How many units of Insulin on average per day? _______Units

2. Are you (or were you ever) treated for **inflammation** of the pancreas (pancreatitis)?

□ No

□ Yes If Yes, in what year? _________

3. Were you ever diagnosed with a **pancreatic cyst** before?

□ No

□ Yes If Yes, in what year? _________

4. Were you ever diagnosed with **pancreatic cancer** (pancreatic carcinoma)?

□ No

□ Yes If Yes, in what year? _________

5. Did you ever undergo **pancreatic surgery**?

□ No

□ Yes

6. Do you smoke?

□ No ***Continue to question 7***

□ Yes ***Continue to question 6a***

□ Not anymore, but I used to ***Continue to question 6a***

6a. How many years of your life did you smoke?____________ year(s)

6b. How many cigarettes on average per day? ________ cigarettes

7. Do you drink alcohol?

□ No ***Continue to question 8***

□ Yes ***Continue to question 7a***

□ Not anymore, but I used to ***Continue to question 7b***

7a. How many glasses on average per day? _________ glasses

7b. Did you ever drink more than 5 glasses on average per day?

□ No

□ Yes If Yes, for how many years? _________

8. Was any of your family members ever diagnosed with **inflammation** of the pancreas (pancreatitis)?

□ No ***Continue to question 9***

□ Yes ***Continue to question 8a***

8a. If yes, which member? (for example: father, niece)_____________________________

8a. How old was this person at the time of diagnosis?___________(years)

9. Was any of your family members ever diagnosed with **pancreatic cancer** (pancreatic carcinoma)?

□ No ***Continue to question 10***

□ Yes ***Continue to question 9a***

9a. If yes, which member? _____________________________

9b. How old was this person at time of diagnosis?___________(years)

10. Was any of your family members ever diagnosed with **breast cancer**?

□ No ***Continue to question 11***

□ Yes ***Continue to question 10a***

10a. If yes, which member? _____________________________

10b. How old was this person at time of diagnosis?___________(years)

11. Was any of your family members ever diagnosed with bowel **cancer** (colon carcinoma, cancer of the large intestine)?

□ No ***Continue to question 12***

□ Yes ***Continue to question 11a***

11a. If yes, which member? _____________________________

11b. How old was this person at time of diagnosis?___________(age in years)

**II. The following questions are about your knowledge of your pancreatic cyst.**

1. Do you know what type of pancreatic cyst you have?
   1. Yes, a benign cyst (no cancer)
   2. Yes, a benign cyst (no cancer) that has the potential to become a malignant cyst (cancer)
   3. Yes, a malignant cyst (cancer)
   4. I have been told, but do not remember
   5. No, I have not been told
2. How large is, according to you, the chance that your cyst will progress to a malignant cyst (pancreatic cancer)?
   1. Very small
   2. Small
   3. Average
   4. Large
   5. Very large
3. Have you searched for extra information on your pancreatic cyst?
   1. No
   2. Yes, through the internet
   3. Yes, through my general practitioner
   4. Yes, through friends and family
   5. Yes, through books and/or magazines or scientific journals

**III. The next questions concern undergoing the future follow-up of your pancreatic cyst.**

1. **Please specify to what extent you agree with each of the following statements:**

a. Future regular follow-up of my pancreatic cyst(s)…

|  |  | **StronglyAgree** | **Agree** | **To some extent** | **Disagree** | **Totally disagree** | |
| --- | --- | --- | --- | --- | --- | --- | --- |
| a | Will reduce my concerns about developing pancreatic cancer. | □ | □ | □ | □ | □ |  |
| b | Will create discomfort in my life. | □ | □ | □ | □ | □ | |
| c | Gives me a sense of certainty. | □ | □ | □ | □ | □ | |
| d | May lead to unnecessary worries. | □ | □ | □ | □ | □ | |
| e | Is a good method to detect cancer in time. | □ | □ | □ | □ | □ | |

b. To what extent…

|  |  | **Not at all** | **Somewhat** | **Rather** | **Very much** |
| --- | --- | --- | --- | --- | --- |
| a | Do the follow-up visits convey you a sense of security? | □ | □ | □ | □ |
| b | Are you nervous before a follow-up visit? | □ | □ | □ | □ |
| c | Do you sleep less well in the week before follow-up? | □ | □ | □ | □ |
| d | Do you postpone plans until after the follow-up visit? | □ | □ | □ | □ |
| e | Do you find regular follow-up burdensome? | □ | □ | □ | □ |
| f | Do the advantages of follow-up outweigh the disadvantages? | □ | □ | □ | □ |
| g | Would you be more worried about your cyst if it was not checked regularly? | □ | □ | □ | □ |
| h | Do you dread the next follow-up visit? | □ | □ | □ | □ |
| i | Would you prefer your cyst to be checked less frequently? | □ | □ | □ | □ |

2. How often would you prefer to have your cyst checked, if it was up to you?

□ Every 3 months

□ Every 6 months

□ Every year

□ Every 2 years

□ Never, I would prefer to stop the follow-up

□ Other, please specify________________________________

3. For how long would you prefer to undergo cyst follow-up?

□ 2 years

□ 5 years

□ 10 years

□ All my life, as long as I am fit to do so

□ Other, please specify________________________________

4. Has your fear for the development of pancreatic cancer changed, now you know that your cyst will be followed?

□ Yes, I worry less

□ Yes, I worry more

□ No, I am just as worried as before I started the follow-up

□ No, I did not worry before, and I do not worry now

□ Other, please specify________________

5. How would you feel if pancreatic cyst follow-up was no longer advised, because the risk of developing pancreatic cancer is too low?

□ I would **not be worried** anymore and no longer wish to be checked

□ I would **still be worried** and ask for follow-up

□ I don’t know

**IV. MRI (Magnetic Resonance Imaging) and EUS (Endoscopic Ultrasound) are both used to visualize pancreatic cysts. The next questions concern your expectations regarding these imaging studies.**

**Even if you did not undergo MRI or EUS (yet), we still ask you to answer the following questions.**

1. Do you worry about undergoing a MRI?

□ Not at all

□ A little

□ Quite

□ Very much

□ I don’t know

2. Do you worry about undergoing an EUS?

□ Not at all

□ A little

□ Quite

□ Very much

□ I don’t know

3. Which investigation do you worry about the most?

□ MRI

□ EUS

□ None of the two

□ Both equally

□ I don’t know

**V. The following questions are meant to evaluate your mental state of mind.**

***Please read each item carefully and choose the answer that comes closest to how you have been feeling in the past week.***

1. a. I feel tense or “wound up”.

□ Most of the time

□ A lot of the time

□ From time to time, occasionally

□ Not at all

b. I still enjoy the things I used to enjoy.

□ Definitely as much

□ Not quite as much

□ Only a little

□ Not at all

c. I get a sort of anxious feeling, like something bad is about to happen.

□ Very definitely and quite badly

□ Yes, but not too badly

□ A little, but it doesn’t worry me

□ Not at all

d. I can laugh and see the funny side of things.

□ As much as I always could

□ Not quite so much now

□ Definitely not so much now

□ Not at all

e. Worrying thoughts go through my mind.

□ A great deal of the time

□ A lot of the time

□ From time to time, but not that often

□ Only occasionally

f. I feel cheerful.

□ Not at all

□ Not often

□ Sometimes

□ Most of the times

g. I can sit at ease and feel relaxed.

□ Definitely

□ Usually

□ Not often

□ Not at all

h. I feel as if I am slowed down.

□ Nearly all of the time

□ Very often

□ Sometimes

□ Not at all

i. I get a sort of anxious feeling like “butterflies in the stomach”.

□ Not at all

□ Occasionally

□ Quite often

□ Very often

j. I have lost interest in my appearance.

□ Definitely

□ I don’t take so much care as I should

□ I may not take quite as much care

□ I take just as much care as ever

k. I feel restless, as if I have to be on the move.

□ Very much indeed

□ Quite a lot

□ Not very much

□ Not at all

l. I look forward with enjoyment to things.

□ As much as I ever did

□ Rather less than I used to

□ Definitely less than I used to

□ Hardly at all

m. I get sudden feelings of panic.

□ Very often indeed

□ Quite often indeed

□ Not very often

□ Not at all

n. I can enjoy a good book or radio or TV show.

□ Often

□ Sometimes

□ Not often

□ Very seldom

*Thank you for filling out this questionnaire.*

*We will send you a link to the next questionnaire after your coming follow-up visit.*

*Yours sincerely,*

*The PACYFIC research team.*

# Appendix C

**Table S2**

Univariable and multivariable ordinal logistic regression analysis for potential characteristics associated with willingness to undergo less frequent surveillance. Outcome measures: ‘not at all’, ‘somewhat’, ‘rather’, ‘very much’.

|  | ***Univariable*** | | |  | ***Multivariable*** | | |  |
| --- | --- | --- | --- | --- | --- | --- | --- | --- |
|  | **Odds ratio** | **95% CI** | |  | **Odds ratio** | **95% CI** | | |
| Age (per 10-year increase) | **1.93** | **1.26 - 2.95** | |  | 1.60 | 1.00 - 2.56 | | |
| Gender |  |  | |  |  |  | | |
| Female | Ref |  | |  | Ref |  | | |
| Male | 1.37 | 0.71 - 2.63 | |  | 1.10 | 0.47 - 2.56 | | |
| Country of residency |  |  | |  |  |  | | |
| Netherlands | Ref |  |  |  | Ref |  | | |
| Italy | **15.98** | **7.02 – 36.37** | |  | **12.00** | **4.53 - 31.81** | | |
| Family history of pancreatic cancer |  |  | |  |  |  | | |
| Not present | Ref |  | |  | Ref |  |  | |
| Present | 0.42 | 0.08 - 2.31 | |  | 0.48 |  | 0.09 - 2.47 | |
| Medical history of cancer* |  |  | |  |  |  |  | |
| Not present | Ref |  |  |  | Ref |  |  | |
| Present | 0.69 | 0.30 - 1.56 | |  | 0.33 | 0.10 - 1.06 | | |
| Gastroenterological symptoms^#^ |  |  |  |  |  |  |  | |
| Not present | Ref |  | |  | Ref |  |  | |
| Present | 0.40 | 0.12 - 1.37 | |  | 0.25 | 0.04 - 1.51 | | |
| Years since first cyst detection (per year increase) | 1.06 | 0.95 - 1.18 | |  | 1.04 | 0.90 - 1.22 | | |
| HADS-anxiety | **1.14** | **1.05 - 1.24** | |  | 1.04 | 0.89 - 1.20 | | |
| HADS-depression | **1.17** | **1.06 - 1.29** | |  | 1.12 | 0.95 - 1.32 | | |

**History with cancer included breast, colon, lung, melanoma, prostate and other. ^#^ Symptoms included abdominal pain, weight loss, fatigue and other. Abbreviations: CI, confidence interval; NA, not applied in the model after applying the backward selection method; Ref, Reference.*
